# Evaluation of a novel lysis-based sample processing method to optimize *Vibrio vulnificus* detecting by loop-mediated isothermal amplification assay

**DOI:** 10.1186/s12866-024-03186-8

**Published:** 2024-01-27

**Authors:** Lei Zhang, Jianfei Liu, Kewei Qin, Chenglin Wu, Hui Ma, Lijun Zhou

**Affiliations:** 1 Central Laboratory, The Sixth Medical Center of Chinese PLA General Hospital, Beijing, 100048 China; 2 The Nursing Department of Chinese PLA General Hospital, Beijing, 100853 China

**Keywords:** *Vibrio vulnificus*, LAMP, Rapid detection, Sample processing, Water-lysis

## Abstract

**Background:**

*Vibrio vulnificus* exists as one of the most serious foodborne pathogens for humans, and rapid and sensitive detection methods are needed to control its infections. As an emerging method, The Loop-Mediated Isothermal Amplification (LAMP) assay has been applied to the early detection of various foodborne pathogens due to its high efficiency, but sample preprocessing still prolongs the complete detection. To optimize the detection process, our study established a novel sample preprocessing method that was more efficient compared to common methods.

**Result:**

Using *V. vulnificus* as the detecting pathogen, the water-lysis-based detecting LAMP method shortened the preprocessing time to ≤ 1 min with 100% LAMP specificity; the detection limits of the LAMP assay were decreased to 1.20 × 10^2^ CFU/mL and 1.47 × 10^3^ CFU/g in pure culture and in oyster, respectively. Furthermore, the 100% LAMP specificity and high sensitivity of the water-lysis method were also obtained on detecting *V. parahaemolyticus*, *V. alginolyticus*, and *P. mirabilis*, revealing its excellent LAMP adaption with improvement in sensitivity and efficiency.

**Conclusion:**

Our study provided a novel LAMP preprocessing method that was more efficient compared to common methods and possessed the practical potential for LAMP application in the future.

## Background

Foodborne pathogens cause millions of human infections yearly, and clinical cases are reported increasingly yearly with high morbidity and mortality [1]. With the change in the human diet, increased intercontinental activities, and environmental changes, foodborne pathogens are seriously threatening global public health [2]. Released data from the World Health Organization (WHO) showed that foodborne bacteria had threatened 600 million people worldwide per year since 2010, which has contributed to 7.5% of global deaths [3]. As one of the important foodborne pathogens, marine *Vibrio* infections have been increasing yearly, and it is crucial to take early preventive and control measures [4, 5]. Among the 12 species of pathogenic *Vibrio*, *Vibrio vulnificus* has gradually shown multiple antibiotic resistance potentials attributed to the abuse of antibiotics in aquaculture production, which has led to a deeply disturbing trend in global *Vibrio* infection [6]. Human infection by *V. vulnificus* usually rapidly develops with atypical symptoms such as fever and hypotension, which causes high mortality in a short time. *V. vulnificus* infections have become the major contributor to the gradual increase in the incidence of bacterial foodborne diseases, which poses a great challenge for their clinical diagnosis and treatment [7–9]. Therefore, it is crucial for their prevention and control at the early infecting stage by improving detecting efficiency and shortening detection time in *V. vulnificus*.

At present, the traditional bacterial culture method, Polymerase Chain Reaction (PCR) method, and ELISA method are commonly used for the detection of foodborne pathogens. Culture-based microbial biochemical analysis and identification method requires enrichment of pathogen, which takes at least 3 days to identify the sample [10]. In comparison, molecular biology-based detection technology is more rapid and sensitive. Methods such as PCR, quantitative Real-time PCR, multiplex PCR, recombinase polymerase amplification, and multiplex-tandem PCR are commonly used [11–14]. In addition, biosensors and immunological methods have also been reported for pathogen detection [15]. However, personnel expertise, testing cost, and precision instruments limit the widespread application of the abovementioned methods.

In 2000, Loop-Mediated Isothermal Amplification (LAMP) was developed by NOTOMI [16]. Optimized from the routine PCR method on the rigorous temperature variation, the LAMP assay developed into an isothermal amplification with the advantages of rapid, simple, and high specificity, which greatly improves the detection efficiency of foodborne pathogens [17]. LAMP has been gradually developed as the rapid pathogen diagnosis method successfully used to detect *Vibrio*, *Staphylococcus aureus*, Novel coronavirus, Hepatitis B virus, and other microorganisms [18–21]. The procedure of sample preprocessing before LAMP assay is very necessary, and protocols including boiling method, phenol/chloroform extraction, and paramagnetic particle method are commonly performed. However, these methods could easily lead to sample contamination attributed to the complicated procedure, which then reduces the efficiency of sample extraction, thus affecting the whole process of LAMP assay by increasing detection time.

Aiming to improve the efficiency of the *V. vulnificus* detecting LAMP assay, a complete procedure including a novel lysis-based sample processing method and an optimized LAMP assay was established and evaluated in this study. Pure culture and artificially contaminated seafood by marine *V. vulnificus* were processed throughout the whole method. Detection results were greatly improved in sensitivity, specificity, and timeliness. After further validation with other common foodborne pathogens, the results indicated that the method built in our study were promising and of great applicating potential in the future.

## Results

### The specificity of LAMP assay

The water-lysis method as a pretreatment method was used to evaluate the specificity of the *V. vulnificus* detecting LAMP assay established in this study. The results were shown in Table 1. Among 35 bacterial strains, all of the *V. vulnificus* (*n* = 11) were detected positive, and the non–*V. vulnificus* (*n* = 24) were all detected as negative by the spin filtration method, boiling method and water-lysis method. There were no false-positive or false-negative reactions, indicating our LAMP assay was highly specific. Compared with the spin filtration method and boiling method, the water-lysis method took only 1 min from sample processing to template DNA extraction, suggesting its advantage in timesaving when applied to LAMP assay.

**Table 1:** LAMP and PCR specificity results via three sample processing methods

Bacterial ID	LAMP				PCR		
	water-lysis	spin filtration	boiling		water-lysis	spin filtration	boiling
FC1671	+	+	+		+	+	+
FC1672	+	+	+		+	+	+
FC1673	+	+	+		+	+	+
FC1679	+	+	+		+	+	+
FC1680	+	+	+		+	+	+
FC3054	+	+	+		+	+	+
FC3063	+	+	+		+	+	+
FC3066	+	+	+		+	+	+
FC3069	+	+	+		+	+	+
FC3072	+	+	+		+	+	+
1H00066	+	+	+		+	+	+
1463	-	-	-		-	-	-
1464	-	-	-		-	-	-
1465	-	-	-		-	-	-
1466	-	-	-		-	-	-
17,802	-	-	-		-	-	-
111 − 012	-	-	-		-	-	-
127-008	-	-	-		-	-	-
133-006	-	-	-		-	-	-
133-008	-	-	-		-	-	-
17,749	-	-	-		-	-	-
90	-	-	-		-	-	-
235	-	-	-		-	-	-
522	-	-	-		-	-	-
695	-	-	-		-	-	-
1059	-	-	-		-	-	-
1.1969	-	-	-		-	-	-
1.1612	-	-	-		-	-	-
1A10009	-	-	-		-	-	-
61	-	-	-		-	-	-
15	-	-	-		-	-	-
95	-	-	-		-	-	-
616	-	-	-		-	-	-
615	-	-	-		-	-	-
1256	-	-	-		-	-	-

### The limit of detection (LOD) of LAMP assay

**Fig. 1:**
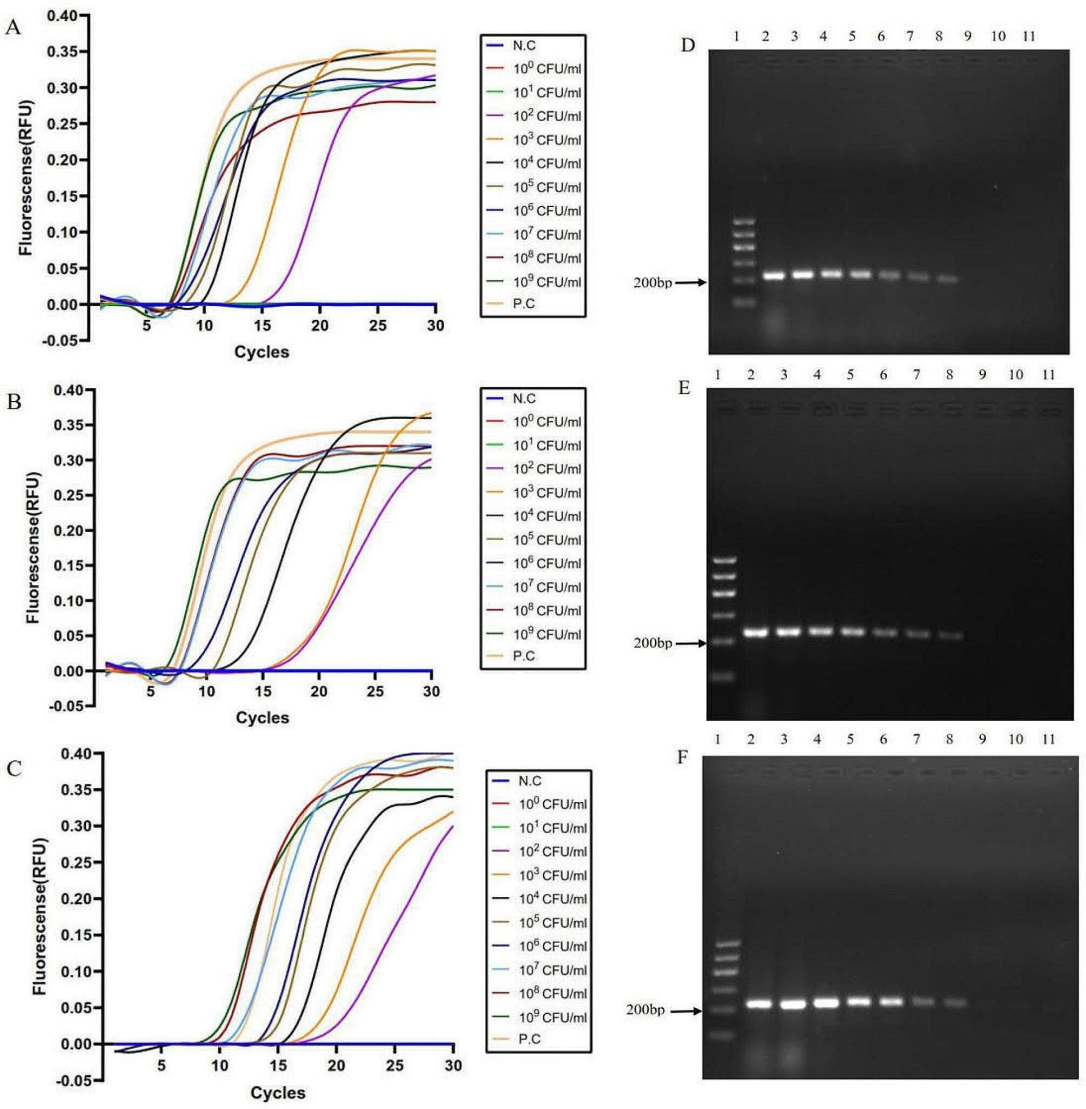
LAMP and PCR sensitivity results via three sample processing methods. (**A**), (**B**) and (**C**) the DNA templates were prepared via the water-lysis, spin filtration and boiling methods respectively, the representative LAMP results showed that *V. vulnificus* 1H00066 presented “S” curves at 10^2^~10^9^ CFU/mL. N.C, negative control; P.C, positive control. (**D**), (**E**) and (**F**) the DNA templates were prepared via the water-lysis, spin filtration and boiling methods respectively, the representative PCR results showed that *V. vulnificus* 1H00066 presented at 10^3^~10^9^ CFU/mL. A band of 233 bp was seen with positive samples. Lane 1 is 100 bp molecular weight marker; Lanes 2 ~ 11, amplification products using 10-fold serial dilutions of a bacterial culture (10^9^ CFU/mL, 10^8^ CFU/mL, 10^7^ CFU/mL, 10^6^ CFU/mL, 10^5^ CFU/mL, 10^4^ CFU/mL, 10^3^ CFU/mL, 10^2^ CFU/mL, 10^1^ CFU/mL and 10^0^ CFU/mL, respectively). The negative control used was water (not shown). In gels, 5 µL of PCR amplicons were loaded per lane

A range of fresh 10× serial diluted *V. vulnificus* 1H00066 was used to evaluate the LOD of the lysis-based LAMP assay established in this study. The results were shown in Fig. 1A, B and C. Using the water-lysis method, spin filtration method and boiling method to extract genomic DNA, *V. vulnificus* ranging between 10^2^ and 10^9^ CFU (Colony-Forming Unit )/mL could be detected by the LAMP assay, along with a negative correlation between fluorescence threshold and the amounts of bacteria. The colony count of *V. vulnificus* culture displayed a LOD of 1.20 × 10^2^ CFU/mL.

When testing the same set of *V. vulnificus* 1H00066 templates by PCR method, the results were shown in Fig. 1D, E and F. Using the water-lysis method, spin filtration method and boiling method separately, the LOD was 10^3^ CFU/mL, which was less sensitive than the LAMP assay. The colony count displayed a LOD of 2.20 × 10^3^ CFU/mL.

**Fig. 2:**
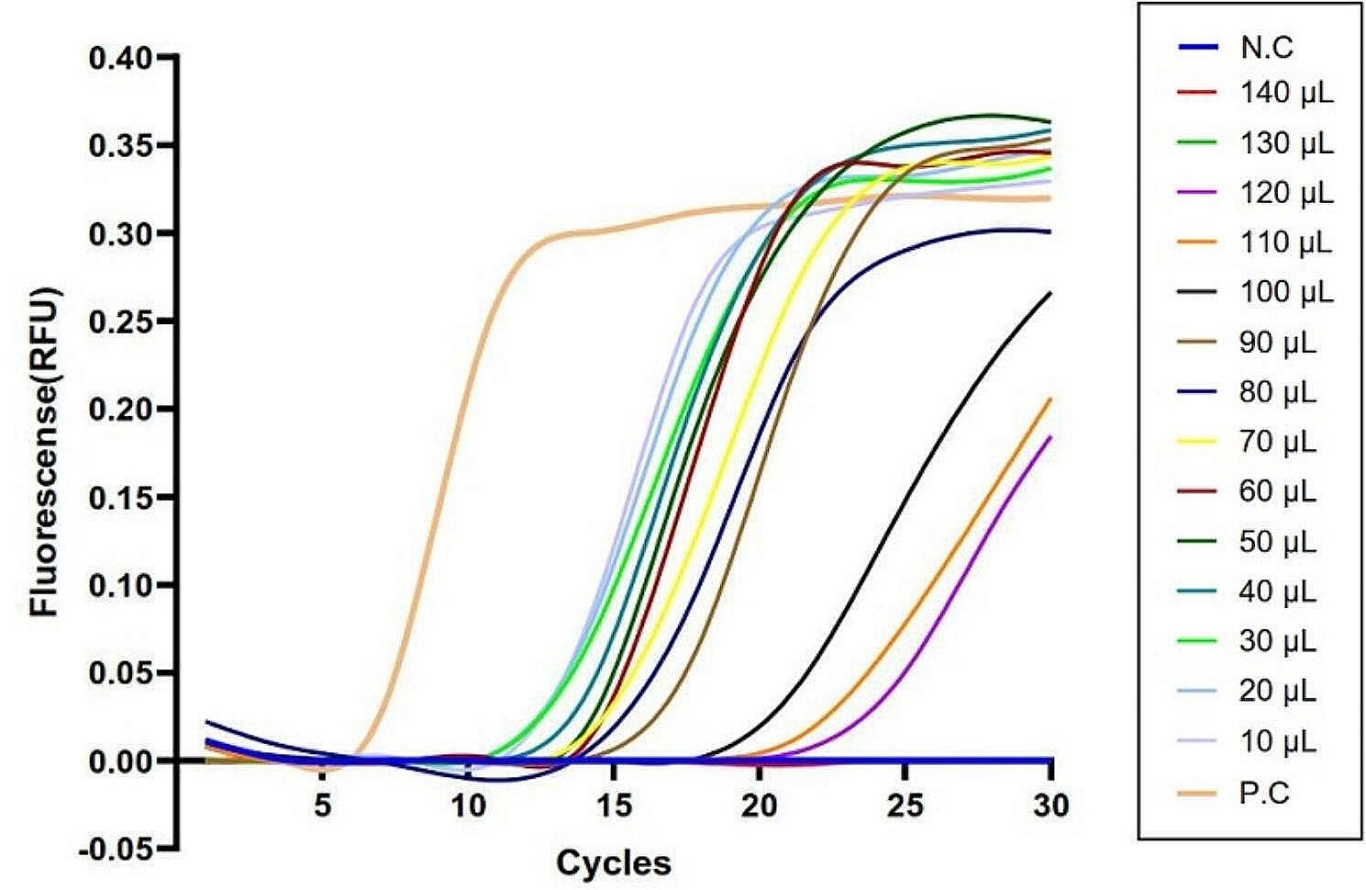
LAMP sensitivity results from different bacteria/water ratios via the water-lysis pre-treating method. Representative results showed that 1.2 × 10^2^ CFU of *V. Vulnificus* 1H00066 was lysed with 10 µL ~ 120 µL of ultrapure water, and “S” curves were obtained via lysis-based LAMP assay; with 130 µL ~ 140 µL of ultrapure water, and straight or slightly oblique lines were obtained via lysis-based LAMP assay. Standard “S” curve, positive result; non-standard “S” curve, positive result; straight or slightly oblique line, negative result; N.C, negative control; P.C, positive control

To further explore the optimal conditions for the water-lysis method as the preliminary processing method applied to the LAMP assay, we obtained different bacteria/water ratios, and the most sensitive bacteria/water ratio was determined. As shown in Fig. 2, positive results were obtained when *V. vulnificus* culture 1.20 × 102 CFU. Lysed in 10 ~ 120 µL of ddH_2_O with a diverse distribution of fluorescence peaks, and the fluorescence thresholds were negatively correlated with bacteria/water ratio. Standard “S” curves were presented when *V. vulnificus* culture lysed in 10 ~ 90 µL ddH_2_O, while non-standard “S” curves were presented when *V. vulnificus* culture lysed in 100 ~ 120 µL ddH_2_O.

### Application of novel lysis-based LAMP assay on artificially contaminated seafood

**Fig. 3:**
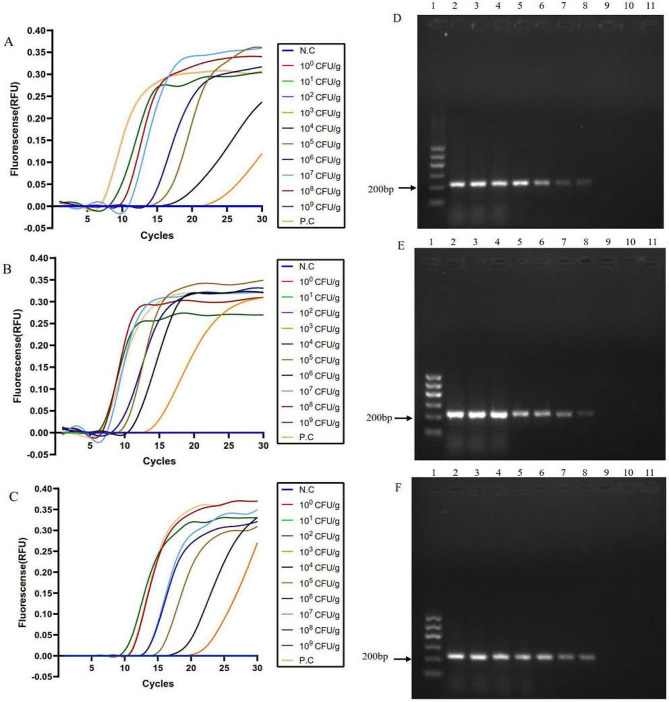
LAMP results on artificially contaminated oyster samples. (**A**), (**B**) and (**C**) the DNA templates were prepared via the water-lysis, spin filtration and boiling methods respectively, the representative LAMP results showed that *V. vulnificus* 1H00066 presented “S” curves at 10^3^~10^9^ CFU/g. N.C, negative control; P.C, positive control. (**D**), (**E**) and (**F**) the DNA templates were prepared via the water-lysis, spin filtration and boiling methods respectively, the representative PCR results showed 10^3^~10^9^ CFU/g. A band of 233 bp was seen with positive samples. Lane 1 is 100 bp molecular weight marker; Lanes 2–11, (10^9^ CFU/g, 10^8^ CFU/g, 10^7^ CFU/g, 10^6^ CFU/g, 10^5^ CFU/g, 10^4^ CFU/g, 10^3^ CFU/g, 10^2^ CFU/g, 10^1^ CFU/g and 10^0^ CFU/g, respectively). The negative control used was water (not shown). In gels, 5 µL of PCR amplicons were loaded per lane

*V. vulnificus* 1H00066 was used to evaluate the practical application of lysis-based LAMP assay in oyster samples, the water-lysis method, the spin filtration method, and the boiling method as preprocessing procedures. The results were shown in Fig. 3A, B and C. Using the abovementioned three methods, sample homogeneous liquid ranging between 10^3^ and 10^9^ CFU/g could be detected by our LAMP assay. Simultaneously, fluorescence thresholds were negatively correlated with artificial contaminating levels. The colony count suggested a LOD of 1.47 × 10^3^ CFU/g.

Further observations were analyzed for positive results. After performing the lysis-based LAMP assay (Fig. 3A), standard “S” curves with stronger fluorescence peaks were obtained on the samples that were contaminated by higher bacterial levels in the range from 10^5^ to 10^9^ CFU/g, while non-standard “S” curves with weaker fluorescence peaks were obtained on the samples that were contaminated by lower bacterial level in the range from10^3^ to 10^4^ CFU/g. In comparison, using the spin filtration method and boiling method to extract genomic DNA from contaminated samples, positive LAMP assay results with standard “S” curves were shown on the samples that were contaminated by bacterial levels in the range from 10^3^ to 10^9^ CFU/g, which all displayed strong fluorescence peaks (Fig. 3B and C).

However, for PCR assays using F3/B3 primers, using the water-lysis method, spin filtration method and boiling method, sample homogeneous liquid ranging between 10^3^ and 10^9^ CFU/g (Fig. 3D, E and **F**), Colony count suggested a LOD of 7.00 × 10^3^ CFU/g for, indicating less sensitive than that of LAMP assays.

### Broad-spectrum application of novel lysis-based LAMP assay

To evaluate whether the water-lysis method was extensively suitable for LAMP assay, two strategies were performed to confirm: detecting the efficiency of strain diversity and LAMP compatibility, respectively.

**Fig. 4:**
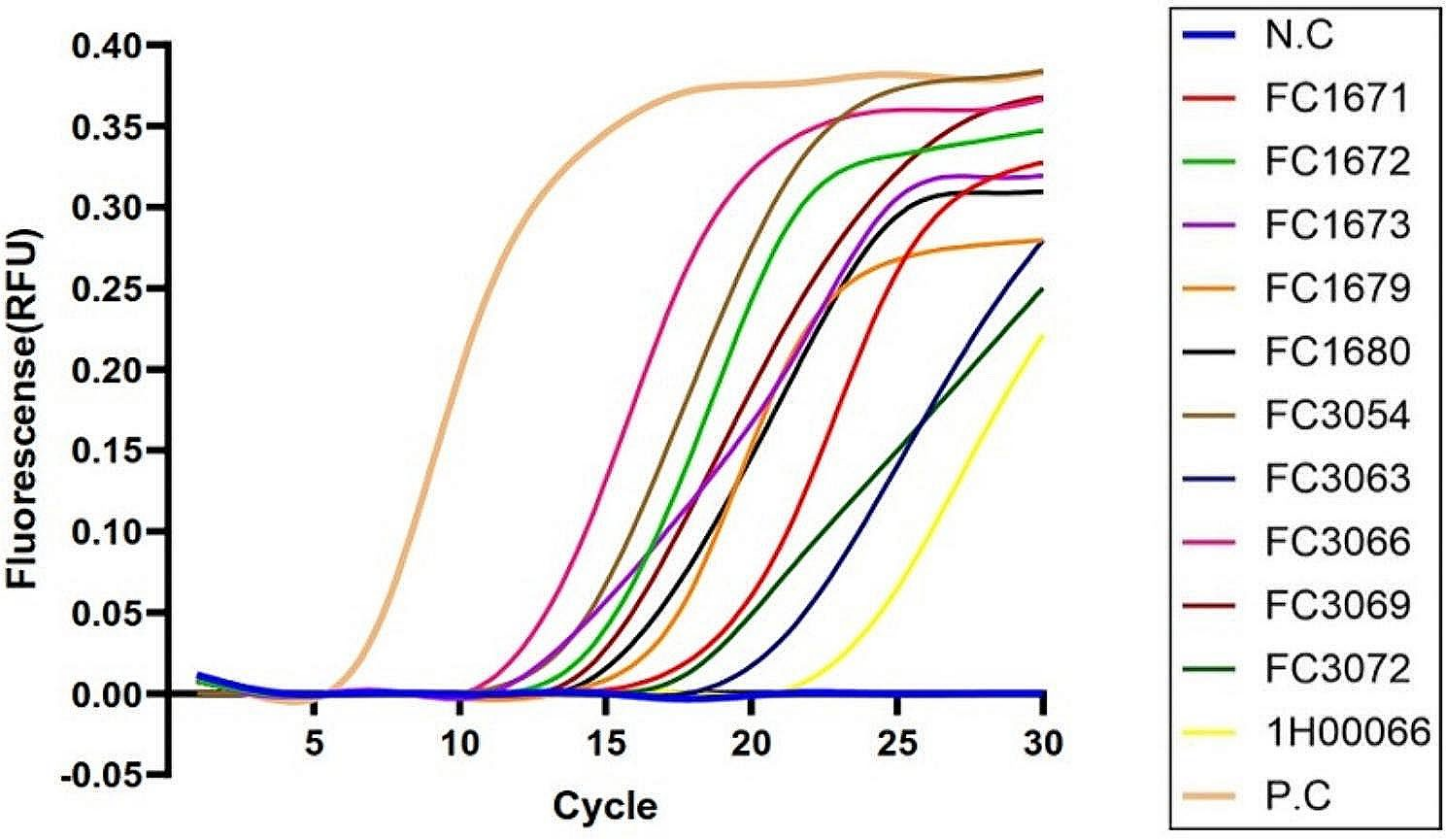
The efficiency of strain diversity of novel lysis-based LAMP assay. 1.2 × 10^2^ CFU of *V. Vulnificus* was lysed with 120 µl of ultrapure water. The representative LAMP results showed that all tested *V. vulnificus* presented “S” curves. Standard “S” curve, positive result; non-standard “S” curve, positive result; straight or slightly oblique line, negative result; N.C, negative control; P.C, positive control

In terms of detecting the efficiency of strain diversity, a total of 11 *V. vulnificus* environmental strains were used as specific detection pathogens, and the lysing system was prepared based on the most sensitive bacteria/water ratio (1.20 × 10^2^ CFU:120 µL) determined above. The LAMP results were shown in Fig. 4. All of the tested strains presented positive results with “S” curves, which included the standard “S” curves (*n* = 8) and non-standard “S” curves (*n* = 3). Meanwhile, the fluorescence peaks distributed in a diverse range with positive results.

**Fig. 5:**
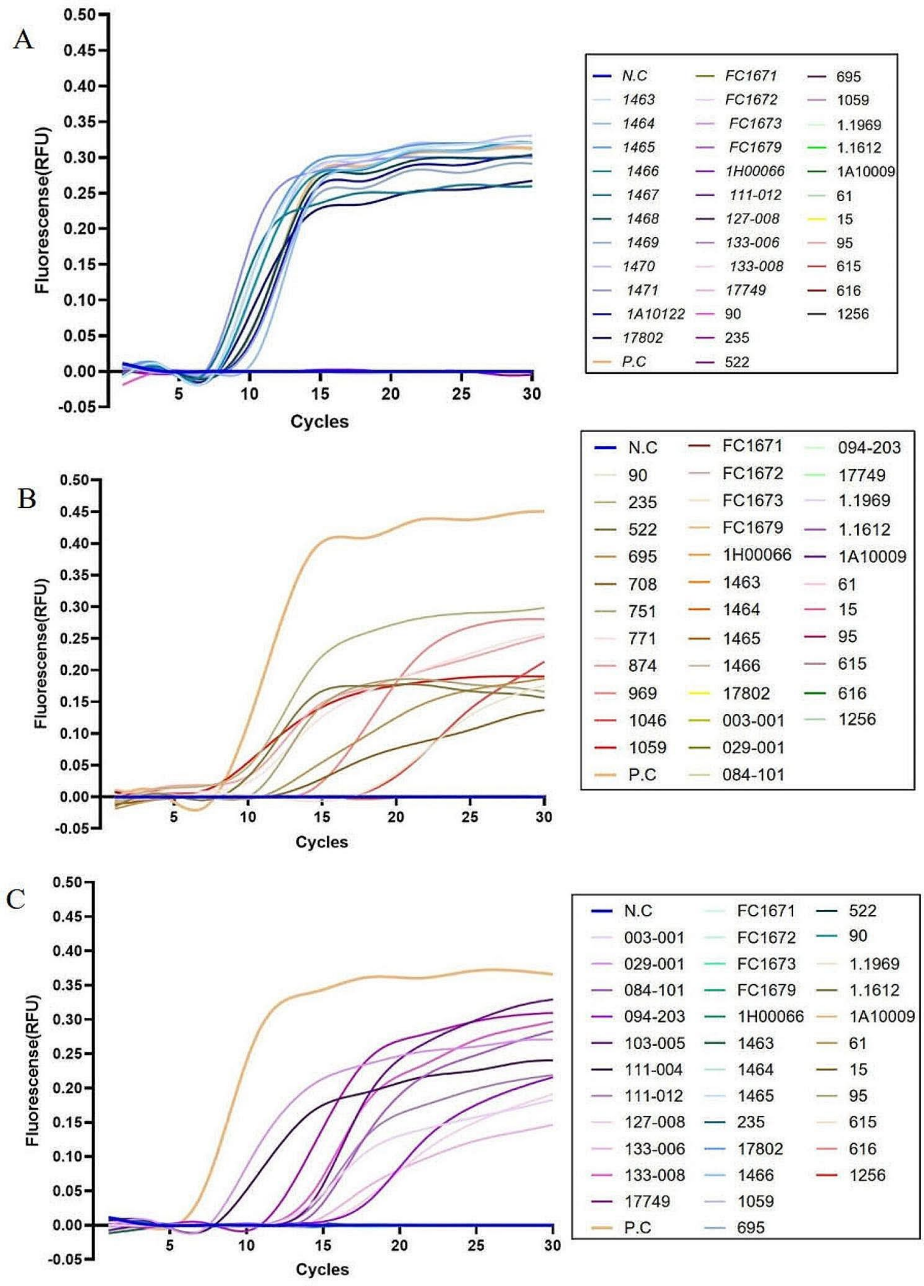
LAMP specificity compatibility of novel water-lysis method as the pre-treating procedure. (**A**) Novel lysis-based LAMP assay on *V. parahaemolyticus* detection and the representative results showed that the detection rate of 11 specific strains was 100%; (**B**) Novel lysis-based LAMP assay on *P. mirabilis* detection and the representative results showed that the detection rate of 11 specific strains was 100%; (**C**) Novel lysis-based LAMP assay on *V. alginolyticus* detection and the representative results showed that the detection rate of 11 specific strains was 100%. Standard “S” curve, positive result; non-standard “S” curve, positive result; straight or slightly oblique line, negative result; N.C, negative control; P.C, positive control

Regarding LAMP compatibility, three commercial LAMP detection kits on *V. parahaemolyticus*, *V. alginolyticus*, and *P. mirabilis* were used, and detecting specificity and sensitivity were evaluated (Fig. 5). Using lysis-based LAMP detection, all results of tested specific strains presented “S” curves, and non-specific strains presented linear amplification, indicating the complete 100% positive detection rates.

In addition, the LAMP compatibility of the water-lysis method was compared within three commercial LAMP detection kits, and *V. parahaemolyticus* was more suitable for its application based on the more standard “S” curves with stronger fluorescence peaks and lower fluorescence thresholds. In comparison, different “S” curves (standard and non-standard “S” curves) were obtained on *V. alginolyticus* (8 standard “S” curves out of 11) and *P. mirabilis* (5 standard “S” curves out of 11), along with diverse fluorescence peaks and fluorescence thresholds.

**Fig. 6:**
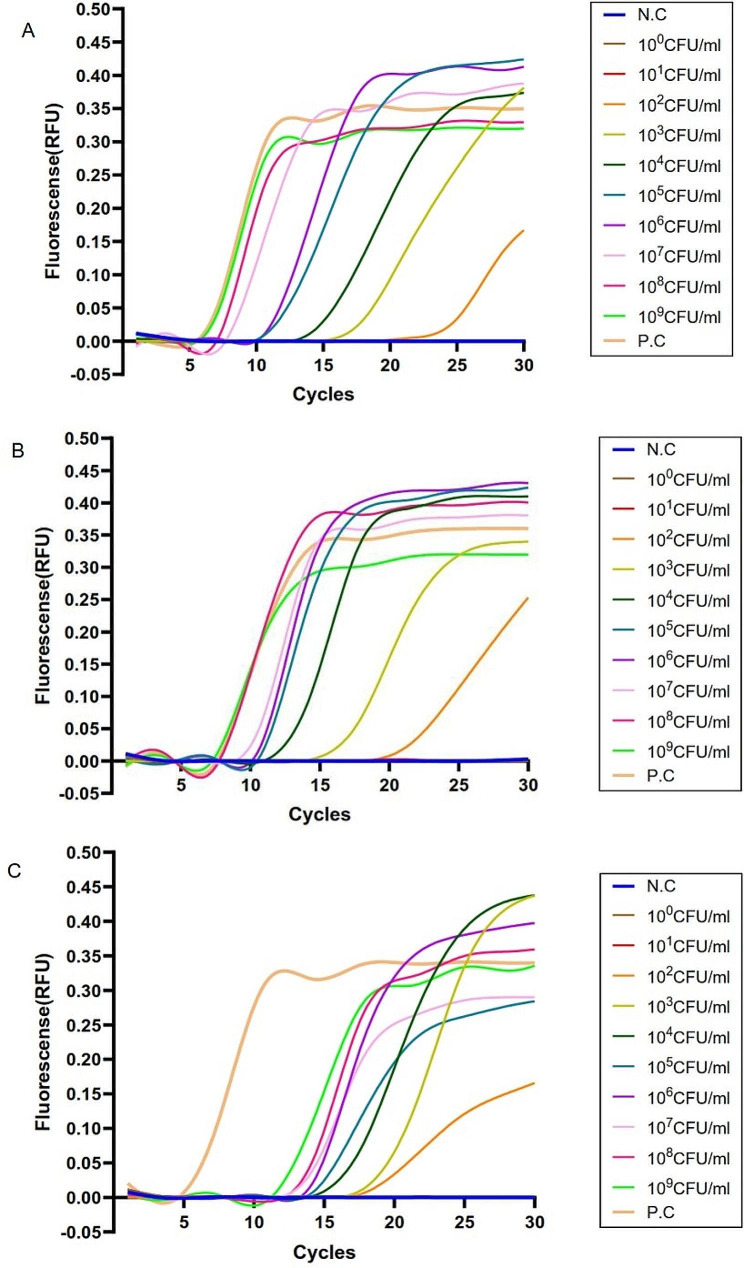
LAMP sensitivity compatibility of novel water-lysis method as the pre-treating procedure. (**A**) Novel lysis-based LAMP assay on *V. parahaemolyticus* detection and the representative LOD was 10^2^ CFU/mL; (**B**) Novel lysis-based LAMP assay on *P. mirabilis* detection and the representative LOD was 10^2^ CFU/mL; (**C**) Novel lysis-based LAMP assay on *V. alginolyticus* detection and the representative LOD was 10^2^ CFU/mL. Standard “S” curve, positive result; non-standard “S” curve, positive result; straight or slightly oblique line, negative result; N.C, negative control; P.C, positive control

During the LOD identification, serial diluted bacteria culture and the minimum bacteria/water ratio were prepared, as mentioned above. The results were shown in Fig. 6. Standard “S” curves were presented on *V. parahaemolyticus*, *V. alginolyticus*, and *P. mirabilis* within the range from 10^3^ to 10^9^ CFU/mL, while non-standard “S” curves could be obtained when the bacteria were as low as 10^2^ CFU/mL, which suggested the diverse positive LAMP results. Colony count suggested that the LOD of *V. parahaemolyticus, V. alginolyticus*, and *P. mirabilis* was 2.25 × 10^2^ CFU/mL, 2.45 × 10^2^ CFU/mL, and 2.10 × 10^2^ CFU/mL, respectively.

## Discussion

Time-consuming and complicated operation are the disadvantages that restrict the use of PCR in field detection of bacteria, and our study aims to solve this problem. In this study, compared with the PCR results, LAMP detection results showed higher sensitivity and shorter time.

As the most important procedure before LAMP amplification, how samples are preprocessed and how genomic DNA is extracted could directly affect the LAMP efficiency and alter the LAMP results. Currently, two sample processing methods are commonly used for LAMP assay based on the published literature. One is the spin filtration method [22]. It needs multiple steps that are time-consuming and easily cross-contaminated for the sample. Another is the boiling method [23], which was used extensively in the earlier literature. The boiling method avoids the disadvantage of time consumption compared with the spin filtration method, yet it still faces the problem of genomic DNA breaks when boiling at high temperatures. It is urgent to develop a more efficient preprocessing method to solve the abovementioned disadvantages when performing LAMP assay. In this study, we developed a convenient sample processing method for LAMP assay, namely the water-lysis method. The pre-treated samples (including pure culture and seafood samples as mentioned above) were only mixed with ddH_2_O at room temperature in an optimized condition, and then the mixture was blended and stood still to obtain genomic DNA for LAMP assay. This water-lysis method was confirmed to improve the efficiency of the LAMP assay by whole procedure evaluation on the specificity, sensitivity, sample type, and commercial applicability. Compared with the boiling method and spin filtration method, the water-lysis method skipped boiling under high-temperature or multi-step operation, which avoided contamination and shortened the whole time of LAMP detection via shortening sample processing time to less than 1 min.

LAMP has already been used in previous papers for *V. vulnificus* rapid detection by targeting a variety of specific genes, such as *gyrB, rpoS*, and *vvha*. Aiming at the *gyrB* gene, Zhou et al. performed a 34 min LAMP detection, and the LOD was 10 fg/µL, indicating high sensitivity and a shorter time than that of other PCR methods for *V. Vulnificus* detection [24]. A *rpoS*-based 90-min LAMP assay was developed by Surasilp et al. reporting a LOD of 1.50 × 10^3^ CFU/mL [25], which was significantly improved in the sensitivity and detection time compared with the traditional PCR method. Due to the specific expression of *vvha* in *V. vulnificus*, it is considered to be another stable biomarker for LAMP detection. From 2008 to 2018, some LAMP assay methods using *vvha* as the targeted gene were established, which gradually shortened the detecting time from 62 min to 35 min and minimized the LOD from 20 CFU/reaction to 4 CFU/tube in pure cultures [26, 27]. In our study, three new pairs of LAMP primers targeting *vvha* were designed, and an optimized 30 min LAMP assay was established. Our results showed that the LOD could be shortened to as low as 120 CFU/mL in pure cultures. Meanwhile, sea product detection results indicated a LOD as low as 1.47 × 10^3^ CFU/g, which suggested its significant in-spot LAMP application potential compared to previous studies using the same targeting gene.

In addition, to optimize *vvha* as the targeting gene, the novel water-lysis method as a sample preprocessing method was evaluated for its application before LAMP amplification. Furthermore, to confirm the broad-spectrum application potential of the water-lysis method for foodborne pathogen LAMP detection, its efficiency of strain diversity and LAMP compatibility were further evaluated, and the water-lysis method reduced sample cross-contamination and shortened detecting time.

Regarding specificity and sensitivity detection, the novel lysis-based LAMP assay showed a 100% accuracy on *V. vulnificus* detection with no false-positive and false-negative results, indicating its high precision. In terms of LOD, the novel lysis-based LAMP assay could be used to detect pure bacterial culture to as low as 1.20 × 10^2^ CFU/mL and seafood samples to as low as 1.47 × 10^3^ CFU/g, which was as sensitive as the results of spin filtration method and boiling method, suggesting its high detecting level of the efficiency. The results of the broad-spectrum application showed that it might be suitable for the water-lysis method to be the pretreatment procedure before LAMP detection on a variety of pathogens with high specificity and sensitivity.

Still, there are limitations in this study. Compared with the results via the spin filtration method and boiling method, non-standard “S” curves presented in a large proportion, and the fluorescence via the water-lysis method varied due to pathogen type, bacterial concentration, and sample type, indicating its stability of the specificity to be improved. The limitations and disadvantages of the novel lysis-based LAMP assay will be further corrected by optimizing pairs of primers and increasing the sample volume. Besides this, the new method for *V. vulnificus* detection will be validated using more aquatic product samples and environmental water samples (including fish, shrimp, oysters, seawater, and river water) from different regions, which will bring this method a step closer to the field. Through these optimizations, lysis-based LAMP assay will provide a method for the rapid detection of *V. vulnificus* in the field.

## Conclusions

This study established a novel lysis-based *V. vulnificus* detecting LAMP assay with great specificity and sensitivity. Through broad-spectrum applicability verification, the water-lysis method built in our study could be applied to several commercial LAMP kits as the sample pre-treating method for foodborne pathogen detection. This lysis-based LAMP assay provided a reference for rapid genomic DNA extraction and early detection of foodborne pathogens, which showed a significant application potential for the prevention and control of foodborne pathogens in the future.

## Methods

### Bacterial strains, growth condition, and seafood materials

A total of 53 bacteria strains were used in this study. As listed in Tables 2 and 1H00066, 1A10122 and 1A10009 were purchased from the Marine Culture Collection of China (MCCC); 1.1969 and 1.1612 were purchased from China General Microbiological Culture Collection Center (CGMCC); strains including ATCC isolates and most of the food, seawater or clinical isolates were generous gifts from other laboratories and preserved in our laboratory. All bacteria were stored at -80 °C and were recovered at 35 °C for 8 h on LB agar plates. The second generation of fresh strains was selected and identified by MALDI-TOF MS. In this research, purified bacterial colonies were isolated and were routinely cultured in LB broth at 35 °C with 200 rpm of shaking for 12 h before preparation.

**Table 2:** Bacterial strains in this study

bacterial genus	bacterial species	strain ID	source
*Vibrio*	*V. vulnificus*	FC1671	Food sample from Beijing province, China
*Vibrio*	*V. vulnificus*	FC1672	Food sample from Beijing province, China
*Vibrio*	*V. vulnificus*	FC1673	Food sample from Beijing province, China
*Vibrio*	*V. vulnificus*	FC1679	Food sample from Beijing province, China
*Vibrio*	*V. vulnificus*	FC1680	Food sample from Beijing province, China
*Vibrio*	*V. vulnificus*	FC3054	Food sample from Beijing province, China
*Vibrio*	*V. vulnificus*	FC3063	Food sample from Beijing province, China
*Vibrio*	*V. vulnificus*	FC3066	Food sample from Beijing province, China
*Vibrio*	*V. vulnificus*	FC3069	Food sample from Beijing province, China
*Vibrio*	*V. vulnificus*	FC3072	Food sample from Beijing province, China
*Vibrio*	*V. vulnificus*	MCCC 1H00066 = ATCC 27,562	Clinical isolates from United States
*Vibrio*	*V. parahaemolyticus*	1463	Food sample from Anhui province, China
*Vibrio*	*V. parahaemolyticus*	1464	Food sample from Anhui province, China
*Vibrio*	*V. parahaemolyticus*	1465	Food sample from Anhui province, China
*Vibrio*	*V. parahaemolyticus*	1466	Food sample from Anhui province, China
*Vibrio*	*V. parahaemolyticus*	1467	Food sample from Anhui province, China
*Vibrio*	*V. parahaemolyticus*	1468	Food sample from Anhui province, China
*Vibrio*	*V. parahaemolyticus*	1469	Food sample from Anhui province, China
*Vibrio*	*V. parahaemolyticus*	1470	Food sample from Anhui province, China
*Vibrio*	*V. parahaemolyticus*	1471	Food sample from Anhui province, China
*Vibrio*	*V. parahaemolyticus*	MCCC 1A10122	Seawater isolates from Mexico
*Vibrio*	*V. parahaemolyticus*	ATCC 17,802	Clinical isolates from Japan
*Vibrio*	*V. alginolyticus*	003 − 001	Seawater isolate from South China Sea
*Vibrio*	*V. alginolyticus*	029 − 001	Seawater isolate from South China Sea
*Vibrio*	*V. alginolyticus*	084–101	Seawater isolate from South China Sea
*Vibrio*	*V. alginolyticus*	094–203	Seawater isolate from South China Sea
*Vibrio*	*V. alginolyticus*	108-005	Seawater isolate from South China Sea
*Vibrio*	*V. alginolyticus*	111-004	Seawater isolate from South China Sea
*Vibrio*	*V. alginolyticus*	111 − 012	Seawater isolate from South China Sea
*Vibrio*	*V. alginolyticus*	127-008	Seawater isolate from South China Sea
*Vibrio*	*V. alginolyticus*	133-006	Seawater isolate from South China Sea
*Vibrio*	*V. alginolyticus*	133-008	Seawater isolate from South China Sea
*Vibrio*	*V. alginolyticus*	ATCC 17,749	Clinical isolates from Japan
*Proteus*	*P. mirabilis*	90	Clinical isolate from China hospital
*Proteus*	*P. mirabilis*	235	Clinical isolate from China hospital
*Proteus*	*P. mirabilis*	522	Clinical isolate from China hospital
*Proteus*	*P. mirabilis*	695	Clinical isolate from China hospital
*Proteus*	*P. mirabilis*	708	Clinical isolate from China hospital
*Proteus*	*P. mirabilis*	751	Clinical isolate from China hospital
*Proteus*	*P. mirabilis*	771	Clinical isolate from China hospital
*Proteus*	*P. mirabilis*	874	Clinical isolate from China hospital
*Proteus*	*P. mirabilis*	969	Clinical isolate from China hospital
*Proteus*	*P. mirabilis*	1046	Clinical isolate from China hospital
*Proteus*	*P. mirabilis*	1059	Clinical isolate from China hospital
*Proteus*	*P. vulgaris*	1256	Clinical isolate from China hospital
*Vibrio*	*V. mimicus*	CGMCC 1.1969 = ATCC 33,653	Clinical isolate from United States
*Vibrio*	*V. furnissii*	CGMCC 1.1612 = ATCC 33,813	River water isolate from Britain
*Vibrio*	*V. fluvialis*	MCCC 1A10009	Seawater isolate from South China Sea
*Escherichia*	*E. coli*	61	Clinical isolate from China hospital
*Citrobacter*	*C. freundii*	15	Clinical isolate from China hospital
*Salmonella*	*S. enteritidis*	95	Clinical isolate from China hospital
*Shigella*	*Sh. flexneri*	615	Clinical isolate from China hospital
*Shigella*	*Sh. sonnei*	616	Clinical isolate from China hospital

Fresh oysters as seafood materials were purchased from the retailing market. The shells were brushed for 10 times successively with double distilled water in their preparation. After the shells were dried, they were sterilized under a UV light for 30 min. The operation procedures involving oysters were completed in 1.5 h in this study, and oysters matching grouping criteria (no *V. vulnificus* detected) were screened following China National Food Safety Standard (GB4789.44-2020).

### Genomic DNA extraction

Genomic DNA was extracted with three methods: the water-lysis method, which was established and emphasized in this study, and two control methods, which were subsequently divided into spin filtration method and boiling method.

The water-lysis method was performed rapidly to obtain genomic DNA templates based on the principle that bacterial cells disruption under the change of osmotic pressure. For the DNA extraction of bacterial culture, 1 mL of prepared culture was centrifuged at 10,000 g for 2 min, followed by the discard of supernatant; after adding ultrapure water (same volume as Tris-EDTA (TE) buffer used in spin filtration method), the solution was mixed for 10 s, standing still for 50 s, After genomic DNA was obtained, final supernatant from the upper layer was used as the DNA template for the subsequent assay.

In the spin filtration method, DNA was extracted using TIANamp Bacteria DNA Kit (Beijing Tiangen Biotechnology Co. Ltd., Beijing, China) and TIANamp Tissue DNA Kit (Beijing Tiangen Biotechnology Co. Ltd., Beijing, China) for pure culture and artificial contamination tissue, respectively. The boiling method used for DNA extraction was performed as Feifei Han described [26].

### Design of LAMP primers

The specific *vvha* gene of *V. vulnificus* (GenBank ID = M34670.1), also known as the hemolysin Vvha encoding gene, was used as the targeting gene in our LAMP assay. Three pairs of primers including one pair of outer primers (F3 and B3), one pair of inner primers (FIP and BIP), and one pair of loop primers (FLP and BLP) were designed based on the 6 specific regions of *vvha* sequence using Primer Explorer V4 software (Fujitsu Limited; http://primerexplorer.jp/e), and the detailed primer information was shown in Table 3. All pairs of primers were synthesized by Beijing SinoGenoMax Research Center Co. Ltd.

**Table 3:** Summary of primer sequences targeting *vvha*

primer	sequence(5’-3’)
F3	TCATATCATCTCCGGTAGC
B3	GAGAAAGTTTAACGCTCTCT
FIP	GACAGTCCTAAACCAGTGAGTTCCGTGGTGATTGATTTGA
BIP	GGCGACGCCTTAGTCAATATCGTTGATTGGGTTGTC
FLP	TCGTCACCAGCAATTTGA
BLP	ATTGTCAGCGATGTCACC

### LAMP reaction

A 25 µL of the complete LAMP reaction system was established in this study, which included 2 µL of prepared DNA template and 1 µL of *Bst* warmStart DNA polymerase (New England Biolabs. Beijing, China) as the amplification starter. The 22 µL of LAMP buffer system contained as follows: 1.6 µM of FIP and BIP each, 0.2 µM of F3 and B3 each, 0.8 µM of FLP and BLP each, 1 M of betaine, 8 mM of MgSO_4_, 1.6 mM of dNTP, and 2.5 µL of 10×Thermo pol Buffer (New England Biolabs. Beijing, China). Meanwhile, positive and negative controls were set with plasmid DNA containing *V. vulnificus vvha* gene and TE buffer, respectively.

LAMP amplification was performed on a Chromo4 real-time PCR device (Bio-Rad Laboratories, Inc. Shanghai, China). The amplification system was set with 2 phases of 30 cycles, which were 30 cycles at 63 °C for 15 s (phase 1) and 63 °C for 45 s (phase 2). The fluorescence signal was collected at the end of phase 2, and the results were analyzed.

### Polymerase chain reaction

PCR assay was performed in a 20 µL-volume system comprising1 µL each of primers F3 and B3, 10 µL of Taq polymerase (TaKaRa, Dalian, China), 1 µL of prepared DNA template and 7 µL of DNase/RNase-Free Water, using the following procedure: Predenaturation at 98℃ for 2 min; 30 cycles of denaturation at 98℃ for 20 s, primer annealing at 55℃ for 20 s; extension at 72℃ for 20 s, and final extension at 72℃ for 5 min. The PCR product was visualized by 2% gel electrophoresis.

### Specificity of LAMP assay

A total of 11 strains of *V. vulnificus* and 24 non-specific strains were pre-treated with the water-lysis method, spin filtration method and boiling method, respectively. DNA templates were used for both LAMP and PCR amplification, preliminary sample treating methods were comparatively evaluated, and specificity was calculated.

### LOD of the LAMP assay

*V. vulnificus* type strain 1H00066 was used to evaluate the LOD of LAMP. The fresh bacteria solution was 10× serial diluted in LB broth for 10 times, and 100 µL of each gradient was prepared for LAMP and PCR amplification. Meanwhile, the LOD of fresh bacteria was calculated as follows: 100 µL of each gradient were inoculated onto LB agar and incubated overnight at 35 °C; the bacterial colonies on the LB agar plates were calculated as the LOD of LAMP and PCR amplification. Two duplicates were counted for each gradient when the LOD was calculated.

Further, to evaluate the bacteria/water ratio (the ratio of bacterial colony number (CFU) and ddH2O volume (µL)) sensitivity when using the water-lysis method as the preliminary treating method for LAMP, a bacteria/water ratio serial dilution was set by adding same bacterial CFU into different volume ddH_2_O(10µL, 20µL, 30µL, 40µL, 50µL, 60µL, 70µL, 80µL, 90µL, 100µL, 110µL, 120µL, 130µL, 140µL). The detecting results were evaluated by the water-lysis-LAMP method.

### Detection of *V. vulnificus* in spiked oysters

For the preparation of artificially contaminated oysters, a 1:10 (oyster: PNCC (Peptone-Sodium Chloride-Colistin enrichment broth, Beijing Land Bridge Technology Co. Ltd., Beijing, China)) homogenate was prepared by homogenizing 25 g of oyster with 225 mL of PNCC for 2 min. One hundred µL of 10-fold serial dilutions of *V. vulnificus* 1H00066 aliquots were inoculated in 900 µL of homogenate and mixed well, followed by centrifuging at 1000 g for 1 min to remove oyster tissues. Then the supernatants were transferred to a centrifuge tube and centrifuged at 12,000 g for 5 min to pellet bacterial cells. DNA template was extracted with three methods as described in the “Genomic DNA extraction” Sect. 2 µL of prepared DNA template was used for LAMP and PCR amplifications.

### Evaluation of the broad-spectrum application of novel lysis-based LAMP assay

The broad-spectrum application of novel lysis-based LAMP assay was evaluated in two aspects. On the one hand, the minimum bacteria/water ratio was prepared in *V. vulnificus* environmental isolates based on the abovementioned procedure, and the complete water-lysis-based LAMP assay was performed so that the potential of the water-lysis method could be evaluated. On the other hand, to evaluate whether the water-lysis method was suitable for LAMP assay on other pathogens, 3 common pathogens, i.e., *V. parahaemolyticus*, *V. alginolyticus*, and *P. mirabilis*, were screened and detected. A total of 3 commercial LAMP assay kits (Guangzhou Double Helix Gene Technology Co. Ltd., Guangzhou, China) were purchased and performed to detect the specificity and sensitivity using the pretreatment procedure mentioned above.

### Statistical analysis

LAMP results were decided based on the fluorescence signal collected and analyzed by Bio-Rad CFX Manager 3 software (Bio-Rad Laboratories, Inc. Shanghai, China). A positive result was identified as an “S” amplification curve was obtained, and a negative result was identified as a linear amplification was obtained. A non-standard “S” amplification curve was considered positive, while a slightly oblique amplification curve was considered negative. All the LAMP assay in this study was repeated three times, and the triplicates were used only when the results were consistent (all shown in “positive” or “negative”).

## Data Availability

All data generated or analyzed during this study are included in this published article.

## Abbreviations

LAMP: Loop-Mediated Isothermal Amplification
CFU: Colony-Forming Unit
LOD: Limit of Detection
PNCC: Peptone-Sodium Chloride-Colistin
TE: Tris-EDTA
PCR: Polymerase chain reaction
